# Relevance of Leg Rehabilitation to Modulating Neurogenic Lower Urinary Tract Symptoms: A Systematic Review

**DOI:** 10.3390/bioengineering12020127

**Published:** 2025-01-29

**Authors:** Gianluca Ciardi, Donatella Giraudo, Milena Fontana, Chiara Citterio, Paola Gandolfi, Gianfranco Lamberti

**Affiliations:** 1 Department of Rehabilitative Medicine, Azienda Usl Piacenza, Fiorenzuola d’Arda Hospital, Via Roma 29, 29017 Fiorenzuola d’Arda, Italy; gianluca.ciardi@unipr.it (G.C.); c.citterio@ausl.pc.it (C.C.); p.gandolfi2@ausl.pc.it (P.G.); 2 Physiotherapy Degree Course, Department of Medicine and Surgery, University of Parma, Piacenza Training Centre, Viale Abruzzo 12 B/C, 29017 Fiorenzuola d’Arda, Italy; milena.fontana@studenti.unipr.it; 3 Department of Urology, San Raffaele Turro Hospital, Via Stamira D’Ancona, 20, 20127 Milano, Italy; giraudo.donatella@hsr.it

**Keywords:** lower limb exercise, neurogenic lower urinary tract dysfunction, neurological disease, rehabilitation

## Abstract

Neurogenic lower urinary tract dysfunction (NLUTD) is a secondary complication of a wide range of neurological disorders, which affects patients’ everyday life and self-efficacy. Some brain imaging studies have shown an overlap between motor activation of the pelvic floor and lower limbs. This systematic review sought to examine the possibility of improving overactive bladder outcomes through a conservative approach based on lower limb training. We conducted a systematic literature review, following the PRISMA guidelines. The following databases were searched: PEDro, PubMed, TRIP, Cochrane Library, EDS base index, Google Scholar, and CINAHL. The PEDro Scale and Cochrane Risk of Bias Assessment Tool were used to assess the overall study quality and sources of bias. A total of 5567 records were retrieved through the systematic search, of which 104 were sought for retrieval; two cohort studies and one randomized controlled trial were finally included. Urodynamics and specific bladder functionality questionnaires showed preliminary evidence of improvement following lower limb stimulation, implemented according to different treatment types (exoskeleton training and weight-suspension walking training). Lower limb-focused exercises showed promising results for improving bladder function, despite the small number of studies and small sample sizes. Future research should confirm this hypothesis using larger samples.

## 1. Introduction

Neurogenic lower urinary tract dysfunction (NLUTD) encompasses a spectrum of urinary disorders resulting from abnormal nervous system control of the bladder [1,2,3]. This condition significantly affects individuals with a variety of neurological impairments other than spinal cord injury (SCI) [4], including multiple sclerosis, Parkinson’s disease, diabetes-induced neuropathy, and other conditions that lead to various degrees of bladder dysfunction [5]. The broad relationship between NLUTD and neurological disorders highlights the complexity of managing affected individuals, as the type and extent of bladder dysfunction of the lower urinary tract, secondary to neurological pathology, varies depending on the site of the lesion, its extent, and the progression of the neurological disease [6]. Symptomatic expression of NLUTD has been hypothesized to be the result of several pathophysiological factors, including modifications of the urethra/urothelium, urinary microbiota, and hormonal/gastrointestinal dysfunctions [7]. Classification schemes for lower urinary tract dysfunction have been proposed [8,9] as follows: suprapontine lesions, which typically present with urinary incontinence secondary to detrusor overactivity, with normal bladder-sphincter synergy [10]; suprasacral lesions, often characterized by bladder-sphincter dyssynergy with urinary incontinence and concurrent partial retention [11]; and sacral lesions with compromised detrusor function and sphincter and pelvic floor muscle paralysis [12]. In suprapontine lesions, lower urinary tract dysfunction may be due to, or strongly influenced by, motor and cognitive dysfunctions, which can cause functional urinary incontinence [13].

In the management of bladder disorders in patients with neurological conditions, a comprehensive clinical assessment, incorporating a thorough integration of clinical data, urodynamic findings, and imaging results, and the formulation of a tailored rehabilitation program are crucial. The initial step of this evaluation involves detailed anamnesis, including gathering information on the onset, history, precipitating factors, frequency and severity of episodes, pharmacological treatments, colonic inertia, and comorbid conditions, such as obesity, diabetes, and previous perineal dysfunction, particularly in female patients [14,15]. Recently, the adoption of quantitative instruments has enhanced the precision of symptom evaluation. Notably, a 36-h bladder diary [16] offers insights into fluid intake and output balance, voiding frequency, and characteristics of incontinence. Furthermore, disease-specific validated scales can quantify the impact of urinary symptoms on patients’ quality of life [17,18]. The subsequent phase entailed a physical examination to identify contributing factors and assess high-risk comorbidities. This includes the evaluation of sacral area sensitivity, anal tone, pelvic sensation, and the bulbocavernosus and anal reflexes [1,12]. Ultimately, urodynamic testing provides a detailed analysis of the mechanism of incontinence and the potential risk of urinary tract damage, warranting periodic assessments to inform patient management effectively [19,20,21,22].

The therapeutic landscape for NLUTD is extensive and encompasses a range of pharmacological and non-pharmacological interventions. Pharmacologically, antimuscarinics and beta-3 adrenoceptor agonists are frequently employed to manage detrusor overactivity by reducing bladder muscle contractions [23,24,25]. For more severe cases, intradetrusor injections of botulinum toxin have demonstrated efficacy in decreasing incontinence episodes by blocking the release of acetylcholine at the neuromuscular junction, decreasing detrusor contractility [26,27].

Beyond pharmacotherapy, neuromodulation techniques have been recognized to have potential for restoring bladder control by directly modulating the neural pathways involved in bladder dysfunction [28,29]. Sacral neuromodulation [30,31] involves electrical stimulation of the sacral nerve roots to restore normal bladder activity, whereas percutaneous tibial nerve stimulation (PTNS) uses electrical impulses delivered to the tibial nerve, leveraging its somatic afferent inputs to influence neural control of the bladder. These approaches offer a less invasive alternative and have yielded promising outcomes.

Exercise can also be considered a neuromodulatory strategy, and its positive effects on neuronal plasticity in cases of disability resulting from neurological diseases are well-known [32]. Similarly, the efficacy of pelvic floor therapeutic exercises has been demonstrated in lower urinary tract disorders after stroke and in cases of multiple sclerosis [33,34,35,36,37,38].

In a cohort of healthy men, a previous study showed that pelvic floor activity activated cortical regions associated with lower extremity movement, suggesting an overlap in the brain areas controlling pelvic functions and those controlling leg movement [39]; in addition, integration seems extended to spinal circuits, as suggested by post-SCI restoration animal models [40]. This finding aligned with those of earlier brain imaging studies, which indicated that stimuli from leg movements could inhibit overactive bladder symptoms, thereby supporting the concept of peripheral neuromodulation. Considering the evidence of an overlap of the motor control areas of the pelvic floor and that of the lower limbs in the brain, it could be hypothesized that it would be possible to achieve a similar control effect secondary to exercises proposed for the lower limbs.

The rationale behind considering lower limb exercise as a neuromodulatory strategy for detrusor overactivity lies in the potential of such physical activity to influence bladder control via neural plasticity. Regular, targeted exercise may not only enhance general health but may also exert a specific modulatory effect on bladder function by engaging the neural mechanisms shared between lower limb and pelvic floor activities.

Despite the extensive neurophysiological background supporting the role of lower limb exercise in the modulation of NLUTD [41,42,43], no systematic review to date has definitively addressed the topic. There is, therefore, a knowledge gap, both in terms of the exercise modalities to be proposed and their administration in terms of timing and frequency of intervention.

By synthesizing current research findings and integrating insights from neurophysiology and rehabilitation science, in this systematic literature review, we evaluated whether lower limb-based exercise, as a viable, non-pharmacological intervention, can suppress detrusor overactivity through a neuromodulatory effect to manage NLUTD across a wide range of neurological conditions. This investigation will not only contribute to a deeper understanding of NLUTD pathophysiology but also open new avenues for innovative, patient-centered therapeutic strategies.

## 2. Materials and Methods

This systematic review was conducted in accordance with the Preferred Reporting Items for Systematic Reviews and Meta-Analysis for Network Meta-analysis (PRISMA-NMA) [44] guidelines and the Cochrane Handbook for Systematic Reviews of Interventions. The search protocol was registered on INPLASY under number 202270099 (https://inplasy.com/inplasy-2022-7-0099/, 29 November 2024) [45].

Our research questions (RQs) were as follows:

RQ1: Is there a relationship between lower limb-centered rehabilitation and the inhibition of NLUTD?
RQ2: Could a specific rehabilitation program be useful to better manage neurological bladder dysfunction?

### 2.1. Search Strategy

PubMed (Medline), the Cochrane Database, Google Scholar, PEDro, the Trip database, the EDS base index, CINAHL, Web of Science, and Scopus databases were systematically searched for English-language articles published from 2018 to 28 February 2024, following the strategy depicted in Supplementary Material S1.

Articles were considered eligible if they agreed with the items defined by the following PICO model:

(P) Participants: Patients with neurological conditions, with a diagnosis of NLUTD
(I) Intervention: Lower limb exercises focused on controlling NLUTD (hypothesized interventions: leg strength/stretching, exercises, therapeutic walking with braces, and exoskeleton training)
(C) Comparison: Placebo/sham treatments, conventional rehabilitation
(O) Outcome measure: NLUTD Assessment Tool scores, urodynamic parameters

We considered eligible randomized controlled clinical trials (RCTs), observational and quasi experimental studies; systematic review were only considered for citation searching, according to the PRISMA model.

Exclusion criteria regarded studies involving children; patients with non-neurogenic NLUTD; patients who underwent a rehabilitation program focused on PTNS (functional electrostimulation), sacral neuromodulation, and pelvic floor muscle training (PFMT); patients who were treated with botulinum toxin or surgery; or where the outcomes were pain-related questionnaires; all publications different from inclusion criteria.

### 2.2. Data Extraction

After removing duplicates, two reviewers independently extracted records through Zotero Software, and then proceeded to the screening for title and abstract in accordance with the inclusion criteria. In cases of disagreement, consensus was reached through discussion with a third reviewer. Includible articles were then selected for full text retrieval; after excluding not retrieved articles, full text reading guided the final inclusion in the last stage of the present review. Data from included studies were exported on a customized Microsoft Excel spreadsheet. The following data were extracted: first author, publication year, aims, design, intervention, sampling, outcome measures, summary of findings, and limitations. Data extraction was finally completed with methodological scores (Pedro and Cochrane tools).

### 2.3. Appraisal

The study quality was independently assessed by two reviewers using the PEDro scale [46]. According to the PEDro scale, the studies were classified as excellent (9–10 points), good (6–8 points), fair (4–5 points), or poor (<4 points). Additionally, the Cochrane Risk-of-Bias tool (RCTS/Observational studies versions as appropriate) was used for bias risk assessment [47]. Any disagreements were resolved by a third reviewer.

## 3. Results

**Figure 1:**
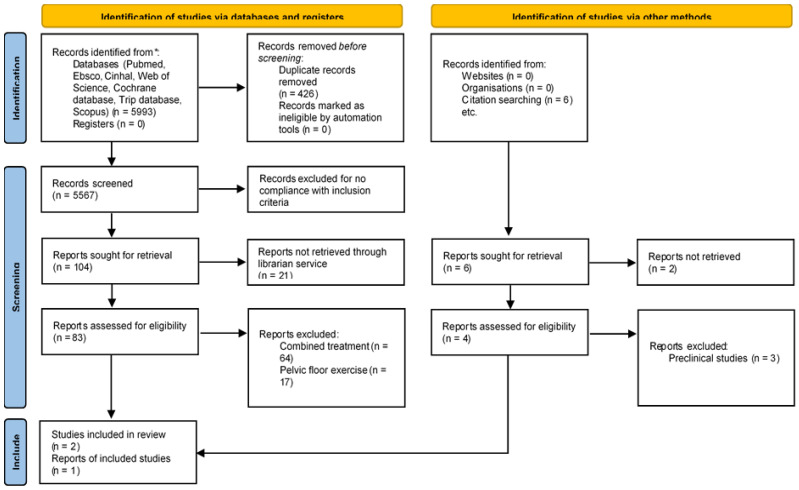
Prisma flow diagram with indication of step-by-step screening of research records.

As described in Figure 1, a total of 5993 results emerged from the overall database search (Supplementary Material S1), of which 426 were excluded after screening for duplicates. The remaining 5567 papers were screened for inclusion according to title/abstract reading, excluding book chapters, conference acts, case series/case reports, animal studies and preclinical models, essays, expert opinion papers, and articles in languages other than English. After this screening, 104 were included for full-text retrieval. Twenty-one studies were not retrieved through our librarian system; finally, 83 papers were assessed for eligibility. Of these, 64 involved combined interventions (including neuromodulation/botulinum toxin/PTNS) and 17 involved PFMT sessions. Finally, two articles were included. Citation searches led to the identification of one more article; therefore, three studies were included in this systematic review.

Two of the included studies were non-randomized double-cohort studies [48,49], while the remaining study was a randomized controlled trial [50]. Two studies [48,49] were conducted by the same author, who had previously carried out similar studies on animals. The data extracted from these studies are reported in Table 1.

### 3.1. Quality Assessment

In terms of methodological evaluation, the PEDro score revealed an overall medium quality of evidence: the scores of the first [48], second [49], and third [50] studies were 5/10, 6/10, and 7/10 points, respectively. The main reason why the included studies did not reach the highest levels of the PEDro scale was the impossibility of applying blinding strategies; given the nature of the interventions, in fact, patients and operators were aware of which treatment was being applied.

A low risk-of-bias emerged based on the Cochrane Risk-of-Bias tool (the observational studies tool for the cohort studies [48,49] and the RCTS tools for the study by Williams et al. [50]).

### 3.2. Synthesis of Results

In the three studies, 40 patients were involved (mean age: 29.5 years, range 19–62 years), of whom 35 were men and 5 were women. A summary of the included studies is presented in Table 1. All studies were conducted in the United States.

Hubscher et al.’s studies [48,49] involved 12 [48] and 22 [49] patients, respectively, with the development of overactive bladder/neurological bowel after SCI. The study by Williams et al. [50] involved six patients with a mean age of 28.5 years (range: 24–49 years), who were at least 6-months post-injury, with SCI at or above the T10 vertebral level. Patients met the requirements for the Ekso and Lokomat devices and had NLUTD.

### 3.3. Intervention

In the first study by Hubscher et al. [48], eight patients underwent 80 (1 h/day) physiotherapy treatments based on treadmill walk training, assisted by a bodyweight support system. Alternatively, locomotor and standing training (weight-bearing without stepping) were performed by the other four patients. Therapists utilized manual facilitation to guide gait training and maximize neuromuscular activity. Treadmill speed was set to a normal walking range (0.89–1.34 m/s) and was varied 25% of the time. In the weight-bearing system training, the participants were instructed to reach and maintain a standing position for 60 min. The remaining four participants in the study received only the usual care.

In the second study by Hubscher et al. [49], 12 participants were selected from the previous study’s standing-only group [48], while an additional 10 participants were selected for inclusion in the arm-training cohort. The first group underwent the same standing training as in the previous study (with a weight-bearing system or customized standing); the remaining group performed 1 h of upper-limb cycle ergometer training, in four to five sessions per week. As patients’ abilities (first endurance) improved, exercise was made more strenuous by increasing resistance.

In the RCT by Williams et al. [50], six participants were randomized to receive either a Lokomat- or Ekso-based walking program. The training intervention included 36–45-min walking sessions of exoskeleton training, three times a week for over 12 weeks.

### 3.4. Outcomes

Regarding bladder outcome measures, all three studies focused on urodynamic examination and specific urinary-referred questionnaires: Hubscher et al. [48,49] used the International Spinal Cord Injury Data Sets Questionnaires–Urodynamics and Lower Urinary Tract Function, whereas Williams et al. [50] used the Qualiveen-30 questionnaire and a bladder diary. Data were collected using a questionnaire administered before and after rehabilitation treatment.

In the two cohort studies [48,49], pre- and post-intervention urodynamics and cystometry were carried out by the same nurse, whereas in the RCT, it was carried out by a blinded assessor and analyzed by a urologist.

Hubscher et al. [48] reported significantly higher detrusorial contractions, lower pressure levels, and an increase in bladder capacity in the lower-limb training group. Furthermore, increases in bladder contraction capacity and detrusor contraction length were also observed. In the second study by this group [49], standing training alone had no significant urological benefits, whereas the upper-limb strengthening group showed significant changes in bladder pressure (lower values) and compliance (higher values), although no change was registered in bladder filling volume or voiding efficacy.

In the third study [50], slight and non-significant benefits in urodynamic examination parameters were observed, probably because of the small number of participants in the standing training treatment.

### 3.5. Lower Urinary Tract Questionnaire and Secondary Outcomes

In terms of secondary outcomes, Hubsher [48] considered the following dataset to investigate bowel, lower urinary tract symptoms, and sexual functions: The International SCI Data Sets Questionnaires for Urodynamics and Lower Urinary Tract Function, Bowel Function, Female Sexual, and Reproductive function/Male Sexual Function. In the second study [49], he further integrated the 15-item International Index of Erectile Function/the 19-item Female Sexual Function Index.

In the study of Williams [50], secondary outcome regarded pelvic floor muscles activation through electromyography, a three-days bladder diary, the SF-Qualiveen questionnaire, speed/walking distance, and effort perception during robotic gait training.

The only significant results for secondary outcome were reported by Hubsher [48,49]: lower urinary tract function dataset questionnaires revealed that nocturia episodes and incontinence were reduced by the end of the training, but the frequency of catheterization remained unchanged [48,49]. In addition, authors reported improvements in fecal incontinence, with fewer referred episodes, a significant decrease in defecation time, and an improvement in sexual desire following treatment; other questionnaires reported only minimal and not significant changes. Williams [50] reported improvements in training related outcomes (speed/distance) and a greater activation of pelvic floor muscles in the Ekso group, while no changes were detected in any of the NLUTD-related questionnaires.

## 4. Discussion

This systematic review investigated whether exercise applied to the lower limbs and changes in neurogenic dysfunction of the lower urinary tract were related. In the three studies included, urodynamics and specific bladder functionality questionnaires provided preliminary evidence of improvement in NLUTD following lower limb stimulation via weight-suspension walking training, and a greater PFM activation following an exoskeleton-based training. The evidence in this systematic review always refers to SCI population, as there was no experience dealing with other NLUTD forms.

To date, a complex model of NLUTD neuro-pathology is being introduced in research [1,51], with symptoms that vary depending on disease localization, evolution, and consequences (so the current NLUTD classification may only serve as a general framework [52]). Central control of micturition has been extensively studied in the past [53]. In the case of suprapontine lesions, two distinct clinical subtypes determining “urgency” are characterized by the presence or absence of detrusor overactivity (DO) [7]. This is particularly important in SCI individuals, where profound alterations of the detrusor afferents occur. As demonstrated in experimental animals, the activation of unmyelinated C-afferent fibers [54,55,56] alters the normal spinal afferents guaranteed by A-delta fibers [57].

Lower urinary tract disorders after SCI represent a severe physical impairment, with a marked psychological impact and adverse effects on quality of life and autonomy, and impose a barrier on social relationships [58,59,60,61]. Rehabilitative therapeutic strategies have always considered motor recovery as central [62], and only after the introduction of intermittent catheterization practice was it possible to observe a reduction in long-term damage to the upper urinary tract [63,64]. To date, the possible consequences of motor exercise on lower urinary tract, intestinal, and sexual dysfunction have not been well studied; in this scenario, locomotor training is classically considered to improve cardiovascular function, strength, and mobility [65,66,67,68,69]. Regarding its effects on NLUTD, several animal studies have demonstrated positive effects of locomotor training [70,71,72]; in humans, the efficacy of exercise on lower urinary tract dysfunction initially appeared as an anecdotal observation and then as a secondary outcome of research using exoskeletons [42], and of epidural stimulation in patients with complete injuries [73]. The hypothesis is based on evidence regarding the effects of PFMT on detrusor function [74,75,76] in individuals with non-neurological and neurological conditions [77,78]. Inhibitory action of PFMT can be induced by the synergistic activity of the lower limb muscles and the pelvic floor itself [75,79], and by connections between different cortical areas involved in pelvic floor activity and lower limb motor areas [39,80]. Based on studies of the overlap of cortical areas controlling the voluntary recruitment of the pelvic floor and some lower limb muscles [81], the effectiveness of locomotor training in incomplete SCI may be determined by the activation of spinal mechanisms induced by muscle afferents [82], in a manner entirely overlapping with the effect induced by stimulation of the tibial nerve [83]. In addition, activating nerve pathways that control coordination of locomotor function could induce adaptive changes in neural networks within the lumbosacral cord, likely including those controlling bladder [84]. Data by our review seems to confirm this hypothesis, with preliminary findings of effectiveness for leg-related exercise on NLUTD control [48,49,50]; despite this, an absolute need to deepen this argument emerged: we do not exactly know neither the optimal dosage of stimulation, nor what is the best form of training to achieve lasting results, nor long-term data (included studies were all pre-post).

In order to better explain the significance of our findings, we conducted a post hoc power analysis using G-Power software [85], using the available data from the Hubscher et al. study, which included 22 patients. The results indicated that the power of the study was low, suggesting that the study may not have been sufficiently powered to detect significant effects of the upper limb strengthening intervention. This limitation should be considered when interpreting the results and highlights the need for future studies with larger sample sizes and adequate power to more accurately assess the intervention’s efficacy. Nevertheless, the preliminary data may provide useful insights for further investigations, as already underlined by precedent literature [64,86].

The results of this review represent a first step toward planning future research and further exploring the role of specific lower-limb exercises in inhibiting an overactive bladder. Future studies should address the current heterogeneity in testing and evaluating the effect of lower limb motor exercises on NLUTD. Researchers should primarily test larger populations, overcoming the current gap in research focusing exclusively on spinal cord injury.

Furthermore, they should study single interventions rather than combinations of several techniques, in order to allow for a true evaluation of their effects. Finally, a higher degree of uniformity is needed in the definition of PROMS (patient-reported outcome measures), as the high variability of datasets hinders standardization and comparability between different studies.

There were some limitations of the present review. First of all, the low number of included papers and low sample sizes only allow us to infer preliminary evidence with respect to the use of lower limb training in the modulation of urinary neurogenic dysfunction. Moreover, studies had different designs and methodologies (two non-randomized cohort studies and one RCT), and this represents an additional difficulty of comparison. Finally, the interventions had the same rationale but were radically different (treadmill or standing weight-supported training, exoskeleton) even in terms of frequency and energy expenditure by the patient.

## Figures and Tables

**Table 1:** Summary of included studies’ findings. Abbreviations: NLUTD: neurogenic lower urinary tract dysfunction; Exp: experimental; Con: control; N = number; LT: locomotor training; UDS: urodynamic study; SCI: spinal cord injury; EMG: electromyography; RPE: rating of perceived exertion; PFM: pelvic floor muscles; SF: short form; RCTs: randomized-controlled trials.

Author, Date	Country	Aim[s]	Design	Intervention	Sampling	Outcome Measures	Summary of Findings	Study Limitations	Methodological Quality
Hubscher et al., 2018 [48]	USA	Identify whether LT as can improve bladder, bowel, and sexual function in chronic SCI (more than two years post-injury) compared to usual cares	Prospective cohort study	12 participants with SCI. Exp (8): 80 session of LT on a weight supported treadmill/LT plus 1 h/day stand training; Cont (4): usual cares prosecution	Convenience sample	UDS; The International SCI Data Sets Questionnaires for Urodynamics and Lower Urinary Tract Function, Bowel Function, Female Sexual, and Reproductive function Male Sexual Function	Significant increase in bladder capacity, voiding efficiency, detrusor contraction time, and decrease in voiding pressure post-training relative to baseline were found in Exp group. Questionnaires revealed a decrease in the frequency of nocturia and urinary incontinence, decrease in time for defecation, increase in sexual desire in Exp group.	Small sample size	Pedro score 5/10 Cochrane rob tool for cohort studies detected a low risk of bias
Hubscher et al., 2021 [49]	USA	Determine if urogenital and bowel improvements in chronic SCI could derive by weight-bearing training or from general exercise	Prospective cohort study	22 individuals with chronic SCI, who were either enrolled in a prior stand training study (N = 12) or upper extremity training (N = 10) Exp: 80 session of standing with weight support Cont: 80 sessions of arm crank	Convenience sample	UDS; International SCI Data Set for lower urinary tract function; International SCI Data Set for bowel function; 15-item International Index of Erectile Function/the 19-item Female Sexual Function Index. All questionnaires were administered pre-/post-training	UDS revealed no significant benefits for Exp group; in Cont group a reduction in bladder pressure and increase of compliance were reported. No differences were identified regarding bowel and sexual functions	Small sample size	Pedro score 6/10 Cochrane rob tool for cohort studies detected a low risk of bias
Williams et al., 2021 [50]	Canada	Testing an exoskeleton-assisted intervention targeting NLUTD in people with motor-complete SCI; compare two exoskeleton programmes	Randomized pilot trial	6 participants Exp (4): 36 sessions of Ekso training Cont (2): 36 sessions of Lokomat training	Convenience sample	Mean speed, distance, and session RPE were taken for each participant during the first and last 5 sessions; EMG for PFM; UDS; 3-day bladder diary; SF-Qualiveen-30.	All participants improved walking speed, distance but not RPE; PFM activity was greater in the Ekso group. UDS, bladder diary, and questionnaires did not clearly change in either group.	Small sample size	Pedro score 7/10 Cochrane rob tool for RCTs detected a low risk of bias

## Data Availability

All data are available within the manuscript.

## Supplementary Materials

The following supporting information can be downloaded at: https://www.mdpi.com/article/10.3390/bioengineering12020127/s1, S1: Search strategy among database.
