# Supplementary material for: Relevance of Leg Rehabilitation to Modulating Neurogenic Lower Urinary Tract Symptoms: A Systematic Review
Source: Bioengineering (Basel). 2025 Jan 29;12(2):127. doi: 10.3390/bioengineering12020127 (PMC11851702; doi:10.3390/bioengineering12020127)
Supplement: Supplementary file 1 [file bioengineering-12-00127-s001.zip › bioengineering-3373534-supplementary.pdf]

DATABASE	SEARCH STRING
Pubmed/Medline	(URINARY BLADDER, NEUROGENIC [MESH] OR NEUROGENIC LOWER URINARY TRACT DYSFUNCTION OR NLUTD) AND (REHABILITATION [MESH] OR LOWER LIMB EXERCISE OR STAND TRAINING OR ACTIVITY BASED TRAINING OR EXERCISE MOVEMENT TECHNIQUES [MESH] OR LEG TRAINING OR EXOSKELETON TRAINING OR EXOSKELETON DEVICE [MESH] OR ROBOT ASSISTED GAIT TRAINING OR RAGT OR ROBOTIC GAIT TRAINING OR PHYSICAL THERAPY MODALITIES [MESH])
Ebsco/Cinhal	(NEUROGENIC URINARY BLADDER OR NEUROGENIC LOWER URINARY TRACT DYSFUNCTION OR NLUTD OR DYSFUNCTIONAL VOIDING OR NEUROGENIC VOIDING DYSFUNCTION OR NEUROGENIC URINARY TRACT SYMPTOM OR DETRUSOR-SPHINCTER DYSSYNERGIA OR NEUR* LOWER URINARY TRACT SYMPTOMS) AND (REHABILITATION OR LOWER LIMB EXERCISE OR STAND TRAINING OR ACTIVITY BASED TRAINING OR EXERCISE MOVEMENT TECHNIQUES OR LEG TRAINING OR EXOSKELETON TRAINING OR EXOSKELETON DEVICE OR ROBOT ASSISTED GAIT TRAINING OR RAGT OR ROBOTIC GAIT TRAINING OR PHYSICAL THERAPY MODALITIES)
Cochrane	(NEUROGENIC URINARY BLADDER OR NEUROGENIC LOWER URINARY TRACT DYSFUNCTION OR NLUTD OR DYSFUNCTIONAL VOIDING OR NEUROGENIC VOIDING DYSFUNCTION OR NEUROGENIC URINARY TRACT SYMPTOM OR DETRUSOR-SPHINCTER DYSSYNERGIA OR NEUR* LOWER URINARY TRACT SYMPTOMS) AND (REHABILITATION OR LOWER LIMB EXERCISE OR STAND TRAINING OR ACTIVITY BASED TRAINING OR EXERCISE MOVEMENT TECHNIQUES OR LEG TRAINING OR EXOSKELETON TRAINING OR EXOSKELETON DEVICE OR ROBOT ASSISTED GAIT TRAINING OR RAGT OR ROBOTIC GAIT TRAINING OR PHYSICAL THERAPY MODALITIES)
Trip database	(URINARY BLADDER, NEUROGENIC OR NEUROGENIC LOWER URINARY TRACT DYSFUNCTION OR NLUTD) AND (REHABILITATION OR LOWER LIMB EXERCISE OR STAND TRAINING OR ACTIVITY BASED TRAINING OR EXERCISE MOVEMENT TECHNIQUES OR LEG TRAINING OR EXOSKELETON TRAINING OR EXOSKELETON DEVICE OR ROBOT ASSISTED GAIT TRAINING OR RAGT OR ROBOTIC GAIT TRAINING OR PHYSICAL THERAPY MODALITIES)
Scopus	ALL (NEUROGENIC URINARY BLADDER OR NEUROGENIC LOWER URINARY TRACT DYSFUNCTION OR NLUTD OR DYSFUNCTIONAL VOIDING OR NEUROGENIC VOIDING DYSFUNCTION OR NEUROGENIC URINARY TRACT SYMPTOM OR DETRUSOR-SPHINCTER DYSSYNERGIA OR NEUR* LOWER URINARY TRACT SYMPTOMS) AND (REHABILITATION OR EXERCISE OR LOWER LIMB EXERCISE OR LEG TRAINING)
Web of science	ALL=((NEUROGENIC URINARY BLADDER OR NEUROGENIC LOWER URINARY TRACT DYSFUNCTION OR NLUTD OR DYSFUNCTIONAL VOIDING OR NEUROGENIC VOIDING DYSFUNCTION OR NEUROGENIC URINARY TRACT SYMPTOM OR DETRUSOR-SPHINCTER DYSSYNERGIA OR NEUR* LOWER URINARY TRACT SYMPTOMS) AND (REHABILITATION OR LOWER LIMB EXERCISE OR STAND TRAINING OR ACTIVITY BASED TRAINING OR EXERCISE MOVEMENT

	TECHNIQUES OR LEG TRAINING OR EXOSKELETON TRAINING OR EXOSKELETON DEVICE OR ROBOT ASSISTED GAIT TRAINING OR RAGT))

Supplementary material 1: search strategy among all included database
